# Progress in Surgery

**Published:** 1897-03-13

**Authors:** 


### Progress in Surgery.

### DISEASES OF THE LARYNX, &c.

(*Continued from page* 383.)

**Abductor Paralysis.**—Some very practical observations are made by Felix Semon and Savery in commenting on a case of bilateral paralysis of the abductors due to malignant stricture of the œsophagus which they report.[9] Whilst more or less incomplete paralysis of both these nerves is of not infrequent occurrence, complete bilateral paralysis is only exceedingly rarely met with, owing to the fact that the death almost always occurs before the lesion has caused the paralysis to be complete. The present case teaches us that, in addition to complete aphonia and dyspnœa, the latter on exertion only, another important symptom may result from the complete paralysis, viz., the impossibility of taking nourishment in the ordinary erect position. The explanation is that when both recurrents are paralysed the closure of the glottis is impossible, and food and drink are therefore apt to pass into the larynx. But the mucous membrane of the larynx is supplied by the internal branch of the superior laryngeal nerve, hence the sensation is not affected, and cough ensues when the food particles enter the larynx. The position recommended by Wolfenden for cases of painful dysphagia was found to be successful, viz., horizontal position on the side, with the head well over the edge of the bed, and fluid nourishment to be taken through a feeding-cup inserted into the lower angle of the mouth. When drinking in this position the fluid passes, not over, but by the side of the larynx through the hyoid fossa, and penetrates into the œsophagus without coming in contact with the posterior surface of the larynx.

**Fibroma.**—Chiari[10] reports a case of an enormous fibroma of the larynx, 2 × 1¾ × 1½ in. being the exact dimension. The patient, a man aged sixty-one, had been suffering for four years with hoarseness and difficulty of breathing, which had much increased during the last four months. It grew from the right aryteno-epiglottidean fold and ventricular band, and during phonation and expiration was pushed right out of the larynx, so as to conceal everything but the epiglottis and arytenoids. It appeared to be soft in consistence, and to be built up of lobes from which little cysts could be seen protruding, pale red in colour, with visible vessels on the surface. It was removed after tracheotomy and insertion of a Trendelenburg's campon-cannula. On cutting the first two rings of the trachea the tumour at once bulged into view, showing it was larger than it had seemed. The tumour was as large as an apple, and was composed of connective tissue, having the structure of a soft fibroma.

**Papilloma.**—Barling[11] has put on record an excellent illustration of the necessity for a guarded prognosis in all cases of apparently funga papillomas occurring in patients past middle age. The patient, now aged 54½ years, was first seen in July, 1892, when thyrotomy was performed, and a large quantity of simple papillomatous growth was removed. Two years later thyrotomy was again performed for recurrence, and the growth then showed distinct, though few cell nests. In July, 1896, thyrotomy was performed for the third time, and the growth removed was a definite epithelioma, but it was entirely confined within the larynx, although suppuration had occurred outside the thyroid cartilages.

**Catarrhal Laryngitis.**—In an instructive paper on unusual forms of catarrhal laryngitis, Rice[12] describes cases of "choked voice," in which condition the ventricular bands become thickened and overlap the true cords, whose functions they seem to replace in some measure. He attributes it to faulty method of using the voice by those who speak or sing in excess. Another condition he calls "congenital vascularity" of the cords, for he has observed able singers whose vocal cords are always bright red. He also refers to a case of a contralto, who could sing acceptably in a church choir with a paralysed left vocal cord.

**Influenzal Laryngitis.**—Compaised[13] records a case of hæmorrhagic laryngitis due to influenza. The patient was a young girl, feverish, aphuric with recurring attacks of cough with hæmoptysis. A pronounced hyperæmia of the pharyngo-laryngeal mucous membrane was accompanied by confluent hæmorrhagic points and large vascular patches on the vocal cord, and by varicosities and hæmorrhagic points on the ventricular bands, interarytenoid space, and arytenoid regions. The patient was cured by appropriate treatment in eighteen days.

**Erythema Nodosum of the Larynx and Trachea.**—C. F. Colt[14] reports a probable instance of this interesting and dangerous condition in a gentleman aged thirty-five. The patient was very dyspnœic, and on making a laryngoscopic examination, mild laryngitis was found, with slight œdema of the false cords, but not sufficient to hide the true cords entirely, which were red and somewhat thickened; the voice was quite clear. The subglottic tissue was plainly visible, but left sufficient room for respiration. Steam inhalations and cocaine had been used for several hours without benefit. Intubation was then tried with various tubes, but an obstruction was always encountered deep down in the trachea. As the patient was rapidly getting worse, it was decided to perform tracheotomy, and he recovered. He had had an attack of rheumatism five years before. In the onset of this last affection he complained of sore throat, following a typical erythema nodosum on the tibiæ and forearms. On the fourth day he had slight dyspnœa, which had passed off; but two days later the second attack, referred to above, came on. The erythema nodosum in the arms and legs went through a typical course, producing successive crops, and this doubtless was the case in the trachea also.

[9] Lanc., Sep. 19, 1896; Jonr. of Laryng., Nov., 1896. [10] Wien Klin. Woch., Aug. 27, 1896; Epit. B. M. J., Oct. 17, 1896. [11] B. M. J., Jan 9, 1897. [12] N. Y. Med. Journ., Oct. 3, 1896. [13] Ann. des Mal. de l'Oreille, May, 1896; Jour, of Laryng., Dec., 1896. [14] Med. and Surg. Rep., Aug. 15, 1896; Jour. of Laryng., Dec., 1896.

### GENITO-URINARY SURGERY.

(*Continued from page* 384.)

Dr. Cabot thinks we have insufficient evidence to determine what are the changes in the prostate which follow castration and lead to a diminution in its volume. The degenerative changes found on microscopic examination in the prostate after castration have also been found in the prostate without castration. The author agrees with Moullin that the improvement which takes place a few hours after the operation is due to diminished vascularity in the gland, brought about by a reflex nerve action. He thinks castration would be especially efficacious in cases of large, tense prostates, when the obstruction is due to pressure of the lateral lobes upon the urethra, but is of little use in myomatous and fibrous prostates. Dr. White, on the other hand, considers that although the softer and more glandular prostates will atrophy more quickly than the very hard, fibrous ones, yet that the latter will also in time atrophy.

Cabot has collected the records of 22 cases of ligature and division of the vas deferens, and the high mortality among these patients shows how many cases die after, but not from, such operations as castration and ligation of the vas. Great improvement occurred in seven cases, and moderate improvement in five, whilst three were not relieved. The author considers it less satisfactory in obtaining relief than castration.

Steinheil[3] has collected records of 57 cases of prostatic hypertrophy in which ligation of the vasa deferentia was performed, and concludes that the clinical results are similar to those of castration. In cases of irregular, hard, fibrous forms of prostatic enlargement, and when the ability of the bladder to contract is completely lost, he prefers prostatectomy.

Dr. David McEwen[4] finds from a study of 37 cases of vasectomy that amelioration occurred in 26, and was sometimes observed as early as the first or second week; the catheter could be passed more easily or urination took place spontaneously, the amount of residual urine decreased, and cystitis was relieved or passed off. Diminution in the size of the prostate was uncertain, and in Guyon's patients was scarcely appreciable.

Mr. Reginald Harrison[5] has had twelve cases of single vasectomy and ten of double. Of the twelve cases of single vasectomy seven derived permanent benefit, and in those of the remaining five who could be traced the result was negative. Out of the ten cases of double vasectomy five were greatly benefited, two were not relieved, and the others were lost sight of and were too recent for any conclusions to be drawn from them as to the value of the operation. The benefit received in the five cases consisted in diminished frequency of micturition or in the use of the cathether; less obstruction to micturition or to the introduction of the catheter, and an improved or normal condition of the urine owing to the bladder being more completely emptied; less liability to hæmorrhage, as, for instance, in connection with the use of the catheter, and decidedly less suffering from prostatic and vesical spasm. Mr. Harrison has had no fatal results after any of these operations. He thinks it best to have an interval of a month between the operations on the two sides. After division of one vas, the prostatic symptoms have subsided temporarily, and then reappeared, as some hypertrophy has taken place in the testicle of the opposite side. He thinks relief of the frequency of micturition can be obtained by the sacrifice of a single vas.

Mr. Mansell Moullin[6] thinks the high mortality of 20 per cent. which he has worked out for prostatectomy is mainly due to performance of the operation when pyelonephritis had set in, and considers that men only a few years over fifty with prostatic hypertrophy, who cannot lead comfortable lives by withdrawing the urine by catheter, should, so long as the urine is still aseptic, have prostatectomy performed rather than castration.

Neinhaus[7] has collected the records of 11 cases of perineal prostatectomy. The rectum is separated from the prostate behind, and much of the gland removed. Spontaneous micturition returned in every case, and continued in all but one, but seven of the cases had persistent fistulæ due to injury of the urethra or rectum. Alexander[8] describes a method of performing perineal prostatectomy which differs somewhat from Nicoll's operation. As in Nicoll's operation the bladder is first opened above the pubes, but the membranous urethra is also opened by a median perineal incision, and through this incision the prostate is enucleated without dividing the mucous membrane of the bladder covering it, the fingers of the operator's other hand pressing the prostate down into the perineum from the interior of the bladder. Alexander began to operate by this method in January, 1894, and has operated on eight cases, with two deaths. The result in the six successful cases was complete restoration of voluntary micturition. He claims the following advantages for the operation—(1) That the entire prostate is thoroughly and immediately removed by enucleation; (2) the mucous membrane of the bladder and postatic urethra is uninjured, and the danger from septic absorption thereby lessened; (3) hæmorrhage is reduced to a minimum; (4) the most efficient and thorough drainage is secured.

**Vesical Stone.**—Freyer, from a consideration of the best methods of removing large calculi from the bladder,[9] comes to the conclusion that Bigelow's method is the safest and best for calculi of all sizes, in patients of all kinds and conditions, provided only that the operation is feasible. When it is not practicable, he thinks that calculi up to about three ounces in the adult and a corresponding weight in the child are best removed by perineal lithotomy, but beyond this size by the suprapubic route. The largest stone he has himself removed by Bigelow's method was six ounces in weight, but he alludes to the fact that Keith, Keegan, and Cunningham in India, and Milton in Cairo, have successfully removed larger stones by the same method. So convinced is he of the vast superiority of Bigelow's method over all others, that he lays it down as an absolute rule, that in no case, whether in the male or in the female, the adult or the child, should a stone, whether small or large, be subjected to a cutting operation until litholapaxy is found to be impossible. Baker,[10] of Hyderabad, calls attention to the fact that stones are occasionally met with which are too hard to be crushed.

De Vlaccos recommends[11] immediate suture of the bladder after suprapubic lithotomy. He thinks it will very rarely be contra-indicated by the condition of the bladder. In only 12 out of 105 cases observed by the author were the changes in the bladder wall such as to render useless the sutures. He advised that a catheter should be left in the bladder, or passed at intervals, and thinks that this will prevent any complication which might otherwise arise from the closure of the wound in the bladder. He uses a continuous catgut suture, and has usually found the bladder-wound healed by the fourth or fifth day.

[3] American Journal of Medical Sciences, Jan. 1897, p. 115. [4] Brit. Med. J., Oct. 10, 1896, p. 989. [5] Ibid. p. 993. [6] Ibid., p. 9[illegible]4. [7] Annals of Surgery, Dec. 1896. [8] New York Med. Record, Dec. 12, 1896. [9] Brit. Med. J., Nov. 7, 1896. [10] Lancet, Oct. 10, 1896. [11] Rev. de Chirurg, Aug., 1895; and B. Med. J. Epitome, Oct. 10, 1896.

